# Ten years of experience with secondary and tertiary cervicofacial lifting: A retrospective study of 360 procedures

**DOI:** 10.1016/j.jpra.2026.02.020

**Published:** 2026-03-01

**Authors:** Tiago G. Reina Di Nunzio, Julien P. Brilmaker, Serge Lê-Huu, Michel E. Pfulg

**Affiliations:** a Laclinic, Montreux, Switzerland; b Clinique de Montchoisi, Lausanne, Switzerland

**Keywords:** Rhytidectomy, Cervicofacial lifting, Aesthetic surgery, Multiple facelifts, Facial aging, Rejuvenation

## Abstract

**Background:**

As society values youth and longevity rises, more patients seek secondary or tertiary facelifts. Published case series on these procedures are rare. This study examines primary, secondary, and tertiary facelift performed by Senior author Dr. Míchel Pfulg over a decade.

**Methods:**

All cervicofacial lifting cases by the Senior author from 2012 to 2022 were reviewed, documenting age, techniques, and concurrent procedures. Complications and longevity across primary, secondary, and tertiary procedures were compared. The author’s surgical technique was outlined.

**Results:**

A total of 256 patients underwent 360 cervicofacial lifting procedures, including 256 primary, 89 secondary, and 15 tertiary facelifts. SMASectomy combined with neck liposuction was the most common procedure. Upper blepharoplasty and lipostructure were the top associated procedures in 31% and 19% of cases, respectively. Complications occurred in 4.6% of cases, with no significant correlation between complication rates and the number of facelifts performed. The average ages for the first, second, and third facelifts were 56, 64, and 67 years, respectively. The average intervals between the first and second facelifts and between the second and third were 13 and 8 years, respectively, with a statistically significant difference.

**Conclusions:**

Secondary and tertiary facelift procedures are increasingly common in treating aging. Understanding the aging process and tissue restoration after surgery is key to achieving optimal results. This study shows that these procedures have low complication rates similar to primary facelifts, although results may not last as long. Achieving consistent and satisfactory outcomes in secondary and tertiary facelifts depends on individualized patient assessment, tailored surgical planning based on previous procedures, and meticulous intraoperative execution.

## Introduction

The anti-aging treatment is, by definition, a treatment designed to reverse the clock. This reverse shift must be accomplished in such a way that the clock restarts ticking thereafter; it has to be consistent with the entire face.^1^ Rhytidectomy has been a procedure that allows us to achieve this objective. According to the statistics of the International Society of Plastic Surgeons,^2^ facelifts have increased by 36% from 2014 to 2018. Also, according to the statistics of the American Society of Aesthetic and Plastic Surgery,^3^ facelifts have increased by 8% from 2019 to 2022. With an aging demographic and interest in maintaining a youthful appearance grows, especially among younger individuals, there has been a rise in the number of patients opting for secondary and even tertiary facelift procedures.^4^

The field of facial rejuvenation surgery has seen significant advancements since its inception in the early twentieth century. Initially, procedures primarily involved removing skin without undermining. Later techniques focused on undermining the skin with closure. However, these skin-based facelift methods often resulted in temporary outcomes and led to the use of the subcutaneous musculoaponeurotic system (SMAS) procedures pioneered by Dr. Tord Skoog.^5^^,^^6^ Surgical techniques that integrate the SMAS have shown to provide longer-lasting and more natural results compared to facelifts that only address the skin.^7^^,^^8^

The distinction between a revision procedure and a secondary or tertiary procedure has been clearly defined by Dr. Sundine.^6^ A revision procedure implies that the initial procedure was not performed in a satisfactory manner and the procedure is being performed to correct inadequacies of the primary operation, whereas a secondary procedure implies that the patient has had an adequate result from the operation but the continuing effects of the aging process and skin laxity have returned and the patient requires another procedure to maintain his or her appearance.

There are several reasons why patients undergo a secondary or tertiary procedure, including the natural aging process, weight fluctuations, lifestyle factors, and patient preferences. While primary face and neck lifts have been extensively examined, secondary and revisional facial rejuvenation procedures have not been as sufficiently discussed in the literature.^4^^,^^9^ There are notable studies focusing on secondary procedures, with the number of studies including tertiary cases being very low. For example, if we search PubMed using the terms “rhytidectomy” or “facelift,” we obtain 3889 results. When we associate “secondary rhytidectomy,” the number of publications is 166, and if we include “tertiary rhytidectomy,” the number of publications decreases to 21.

In this study, a comprehensive examination is conducted of primary, secondary, and tertiary cervicofacial lift cases performed by the Senior author (Dr. Míchel Pfulg) over the past decade. The aim is to describe the surgical technique commonly employed by the senior author, assess the efficacy of secondary and tertiary facelift in rejuvenating the patient’s appearance, taking into account the quantity and nature of complications, and analyzing the long-term effectiveness of procedures both between primary and secondary lifts and between secondary and tertiary lifts.

## Patients and methods

After receiving institutional review board approval, we reviewed a retrospective study where all cases of cervicofacial lifting from 2012 to 2022 performed by the Senior author, Dr. Michel Pfulg, at Laclinic, Montreux, Switzerland, were included. The PulseMedica and Skalpell databases were utilized, where all attentions and procedures conducted during that period were recorded. Secondary and tertiary lifting instances were considered whenever both preauricular and temporal flaps were elevated, thus excluding patients in whom the technique originally described by the Senior author, “The Soft-Lifting,” was carried out.^10^ All patients who underwent cervicofacial lifting by the Senior author were included, regardless of whether any of the three procedures was performed by another surgeon.

A thorough chart review included information from medical histories, including age at each procedure, lifting technique used and the number of pre and postoperative consultations. Other aesthetic procedures performed during the same surgical timeframe, such as lipofilling, blepharoplasties, brow lift, rhinoplasty, and genioplasty, were recorded. Additionally, the quantity and type of complications, such as hematoma, seroma, nerve injury, skin slough, infection, pixie ear and the need for revision, were documented.

The longevity of the treatment between primary, secondary, and tertiary cervicofacial lifting was compared, as well as the frequency of complications in each group using Fisher’s exact test (Software: GraphPad Prism 10.2.0).

## Operative technique

The operative technique most frequently used by the Senior author involves^1^ evaluating the skin laxity, position of the hairline in the temporal, retroauricular, and occipital regions, and the vectors of pull resulting in shifting of the hairlines to plan the incision in each individual patient. In all cases, particular attention was given to preserving the hairline, especially in the temporal region, to minimize visible scarring and avoid hair loss. The oblique incision technique utilized by the Senior author is designed to preserve hair shafts, allowing for regrowth through the scar. This approach helps maintain the natural positioning of the hairline, avoiding the backward shift often associated with traditional facelift incisions. By carefully planning incisions in the temporal and retroauricular regions, hairline displacement is minimized, contributing to improved aesthetic outcomes. The dissection is initiated with liposculpture of the neck. The dissection then starts in the postauricular area with scalpel under direct vision to continue to the neck and the platysma muscle when it becomes blunt, cautiously spreading the scissors. Elevation of the skin in the preauricular region begins. When the dissection is 2 cm in front of the ear, the flap is elevated cautiously spreading the scissors and in the temporal area the flap is torn off by blunt finger dissection. The dissection can be done high enough to slightly raise the eyebrows and correct the lateral canthal region. The dissection of the lateral cheek area and over the zygoma is then performed. The level of SMAS resection is precisely marked with Bonnie’s blue ink. It is horizontal over the zygoma, oblique over the parotid gland, and vertical along the anterior border of the platysma muscle. The vector of elevation of the SMAS is purely vertical. Two key sutures performed with 3-0 PDS are crucial at this stage. The first stitch is to fix the fascia at the point of the maximal vertical elevation of the SMAS over the zygoma, approximately 4 cm in front of the ear. The second suture grasping the platysma muscle about 2 cm lower than the tip of the earlobe and attaching it very strongly to the Loré fascia at the level of the incisura intertrágica. A running suture of 3-0 Vicryl is used to close the fascia. A tube suction drain is always kept in the cheek, extending to the neck before skin closure. Two key sutures are placed: the first and most important stitch is above the ear at the anterior crus of the helix. The second is at the peak of the postauricular incision. Between the sutures, the excess skin is excised in a line parallel to the adjacent skin edges. The skin is closed with 4-0 Prolene in a running intradermal fashion. Cold packs are applied over the neck and preauricular areas in the first 24 hours, changed every two hours. The drains are removed on the first postoperative day, and the patient is discharged.

In secondary and tertiary facelifts, scar tissue and altered tissue quality require special care. Skin elevation must be cautious, leaving 5–8 mm of subdermal fat to preserve vascularization and avoid necrosis. Since the SMAS becomes thinner after repeated lifts, its management should be individualized: SMAS plication is safer in cases of scarring or fragile tissue, while SMASectomy remains an option when feasible. Sharp dissection is preferred in scarred areas to ensure precision and minimize trauma. Careful evaluation and meticulous planning are essential to maintain safety and achieve effective results.

## Results

A total of 256 patients were included in the sample, accounting for 360 cervicofacial lifting procedures over the 10-year study period. Of these, 256 were primary, 89 were secondary, and 15 were tertiary facelifts. Among the patients, 243 (95%) were female, and 13 (5%) were male. The age range varied from a minimum of 39 years to a maximum of 83.

Regarding the total facelifts in the sample, the Senior author performed 225 out of 256 (88%) primary facelifts, 88 out of 89 secondary facelifts, and 14 out of 15 tertiary facelifts. Among the secondary procedures, 58 (65%) were performed on patients whose primary facelift was also conducted by the Senior author. Of the tertiary facelifts, 14 out of 15 (93%) were performed on patients whose secondary procedure had been conducted by the Senior author.

The most frequently employed procedure across all stages (primary, secondary, and tertiary facelifts) was SMASectomy, accounting for 288 (88%), combined with neck liposuction in 105 cases (32%) [Supplementary documents—Table 1].

Out of the patients, 207 (63%) underwent an additional aesthetic surgical procedure in conjunction with their cervicofacial lifting, with upper blepharoplasty being the most commonly associated procedure in 103 cases (31%), followed by lipostructure in 63 cases (19%) [Supplementary documents—Table 2].

The most common preoperative consultations involved two main stages: initial planning and confirmation, in addition to consultations with an anesthesiologist. Postoperative consultations were most commonly scheduled at 24 hours, 6 days, 12 days, and 3 months.

Fifteen complications were recorded from the total procedures, representing a 4.6% incidence. Four cases involved hematoma, two had seroma, all resolved through puncture and drainage. Five patients experienced infection, managed with oral antibiotic therapy. Two cases required revision due to pixie ear, one patient underwent revision for early skin slough, and one patient had zygomatic nerve injury with complete spontaneous recovery two months post-surgery [Supplementary documents—Table 3].

The relationship between the number of complications and the type of facelift (primary, secondary, or tertiary) was not considered statistically significant (*p*-value = 0.6091), indicating no correlation between the number of complications and the number of facelifts performed on a patient.

The average age for the first cervicofacial lifting procedure was 56 years, for the second lifting, it was 64 years, and for the third, it was 67 years. In terms of longevity, the average time between the first and second facelift was 13 years, while the average time between the second and third facelift was 8 years. This difference was deemed statistically significant (*p*-value = 0.004), suggesting that the durability of the first facelift is expected to be greater than that of the second.

## Discussion

Given the increasing prevalence of cervicofacial lifting procedures performed at younger ages, it’s not uncommon to encounter patients seeking second or even third facelifts. Many of these patients were satisfied with the results of their initial facelift but seek further enhancement.

In our patient series, the most frequently used procedure at all stages (primary, secondary, and tertiary facelifts) involved SMASectomy combined with neck liposuction. However, secondary or tertiary facelift can vary significantly from primary surgery.^11^ In secondary or tertiary facelift procedures, there are alterations in anatomy across all layers of dissection. Skin elasticity decreases, while skin thickness reduces, rendering tissue mobilization more complex and hazardous, especially in cases where SMASectomy has been previously undertaken.^12^^,^^13^ Although the principles of secondary or tertiary facelifts resemble those of primary ones, sequelae of the primary procedure such as asymmetry, pixie ear lobe, tragal abnormalities, lateral sweep, hairline malposition, and abnormal scarring, may add to the complexity of the procedure.^14^^,^^15^ Knowledge of the patient’s previous operation is advisable but not always available. Skin redraping without tension and with proper vectoring is critical.^16^

Secondary and tertiary facelifts present unique challenges due to scar tissue and reduced skin elasticity. To address these complexities, we highlight the importance of tailored surgical strategies. Techniques such as sharp dissection in scarred areas, preserving 5–8 mm of subdermal fat during skin elevation, and adjusting SMAS interventions to the patient’s specific anatomy are critical to reducing risks. While this study demonstrated the efficacy of SMASectomy, alternative approaches, including SMAS plication or deep plane facelift, could offer additional benefits, particularly in cases with significant scarring or compromised tissue quality. Comparative studies are needed to better understand the relative advantages of these methods, particularly in secondary and tertiary facelifts.^7^^,^^9^

The facelift procedure, even when incorporating treatments for the brow, eyelids, and neck, does not target the perioral region or address the overall skin texture. Supplementary procedures are necessary to achieve comprehensive facial rejuvenation in these specific areas.^17^ Skin resurfacing lasers, fat grafting, liplift, injectable fillers, and botulinum toxin are commonly used treatments in conjunction with facelifts at all stages. All of these procedures were performed with similar frequency in primary, secondary, or tertiary procedures.

Regarding complications, similar to Dr. Beale’s description,^4^ this study confirms the assertion that secondary and tertiary facelift is a safe and effective surgical procedure for facial rejuvenation in aging patients. Other authors, such as Dr. Funk and Dr. Sundine, found no statistically significant difference in the number of complications between secondary and primary facelifts, with a reported complication rate of 5%. As for the risk of nerve damage, in this case series, an incidence of 0.3% was found, with only one patient experiencing injury to the zygomatic nerve during their primary facelift, and no such complications reported in secondary and tertiary procedures in the series. The incidence of nerve damage reported in different studies ranges significantly, from 2%^4^, ^5^, ^6^, ^7^, ^8^, ^9^, ^10^, ^11^, ^12^, ^13^, ^14^, ^15^, ^16^, ^17^ to 11%, particularly following secondary facelifts involving sub-SMAS dissection.^6^ It seems that techniques such as SMASectomy or SMASplication might offer a safer alternative in secondary facelifts. However, a direct comparison between these techniques and sub-SMAS elevation in the context of secondary facelifts is lacking.^4^

Regarding the duration of results between the first and second facelifts in the literature, it varies, with reported durations ranging from 5 to 12 years.^6^, ^7^, ^8^, ^9^, ^10^, ^11^, ^12^, ^13^, ^14^, ^15^, ^16^, ^17^, ^18^ There are few studies that have compared the longevity of the primary procedure with respect to the secondary procedure, and of the secondary procedure with respect to the tertiary one.^4^ In our series, the longevity of the primary procedure averaged 13 years, while that of the secondary procedure averaged 8 years, with this difference being statistically significant (*p*-value = 0.004). This can be attributed to the fact that the average age of patients undergoing primary facelift in our series was 56 years, while for secondary facelifts, it was 64 years. It’s reasonable to expect that younger patients may have better skin quality, allowing the SMAS to maintain tension effectively over time, resulting in a more consistent appearance. Conversely, in secondary or tertiary facelifts, the anatomy has already been altered by the primary procedure, leading to reduced skin elasticity and thickness. If SMASectomy was performed during the primary procedure, mobilizing tissues becomes more difficult and risky (additional preoperative and postoperative photographs of patients before and after their second and third facelift procedures are shown in Figure 1, Figure 2 and Supplementary documents).

**Figure 1:**
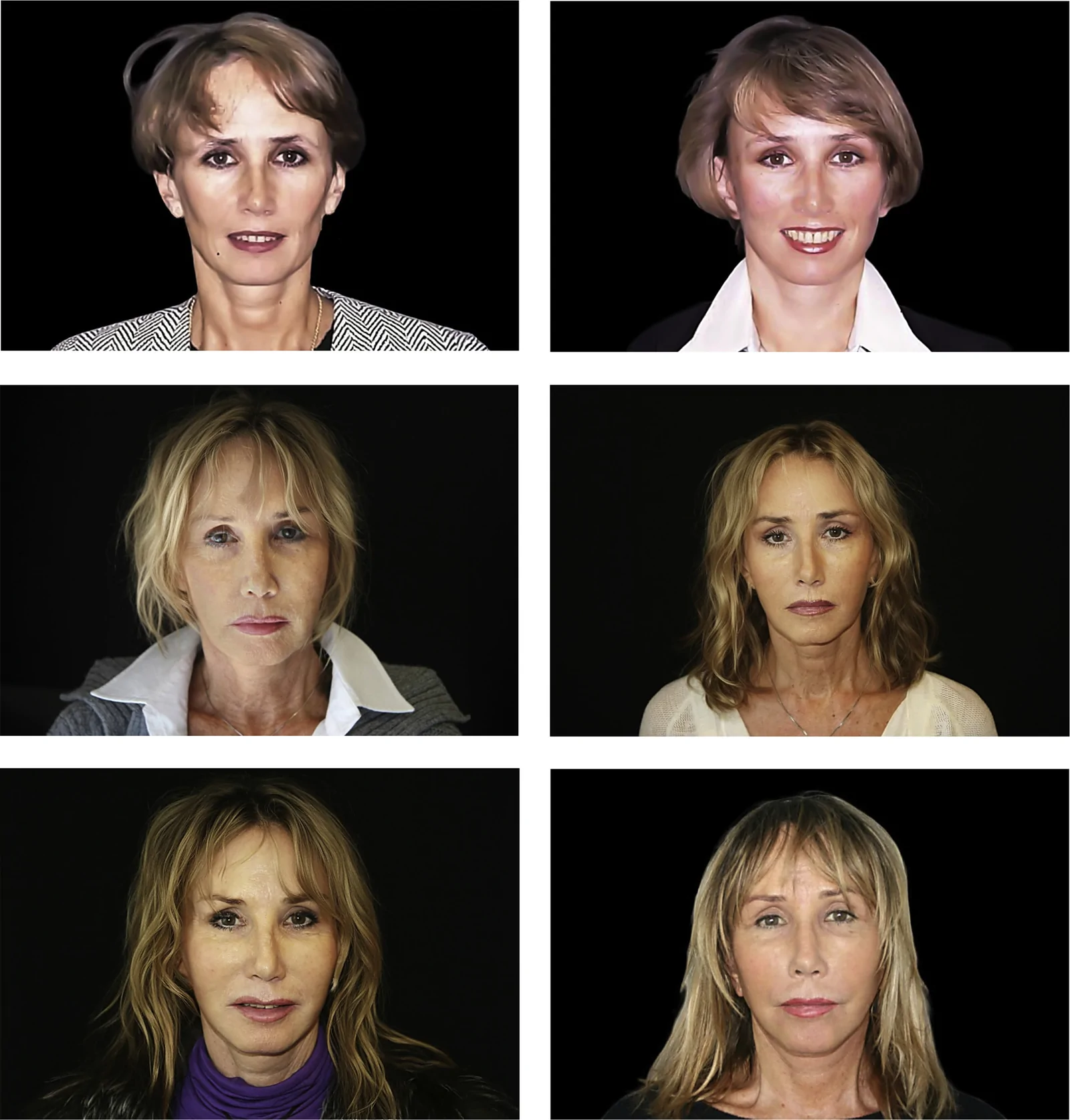
Case 1. Row 1: Age 54, preoperative and 12 months postoperative primary cervicofacial lift combined with brow lift. Row 2: Age 62, preoperative and 12 months postoperative secondary cervicofacial lift and mini-liposuction of the left jawline. Row 3: Age 73, preoperative and 12 months postoperative tertiary cervicofacial lift with lower blepharoplasty and lipofilling.

**Figure 2:**
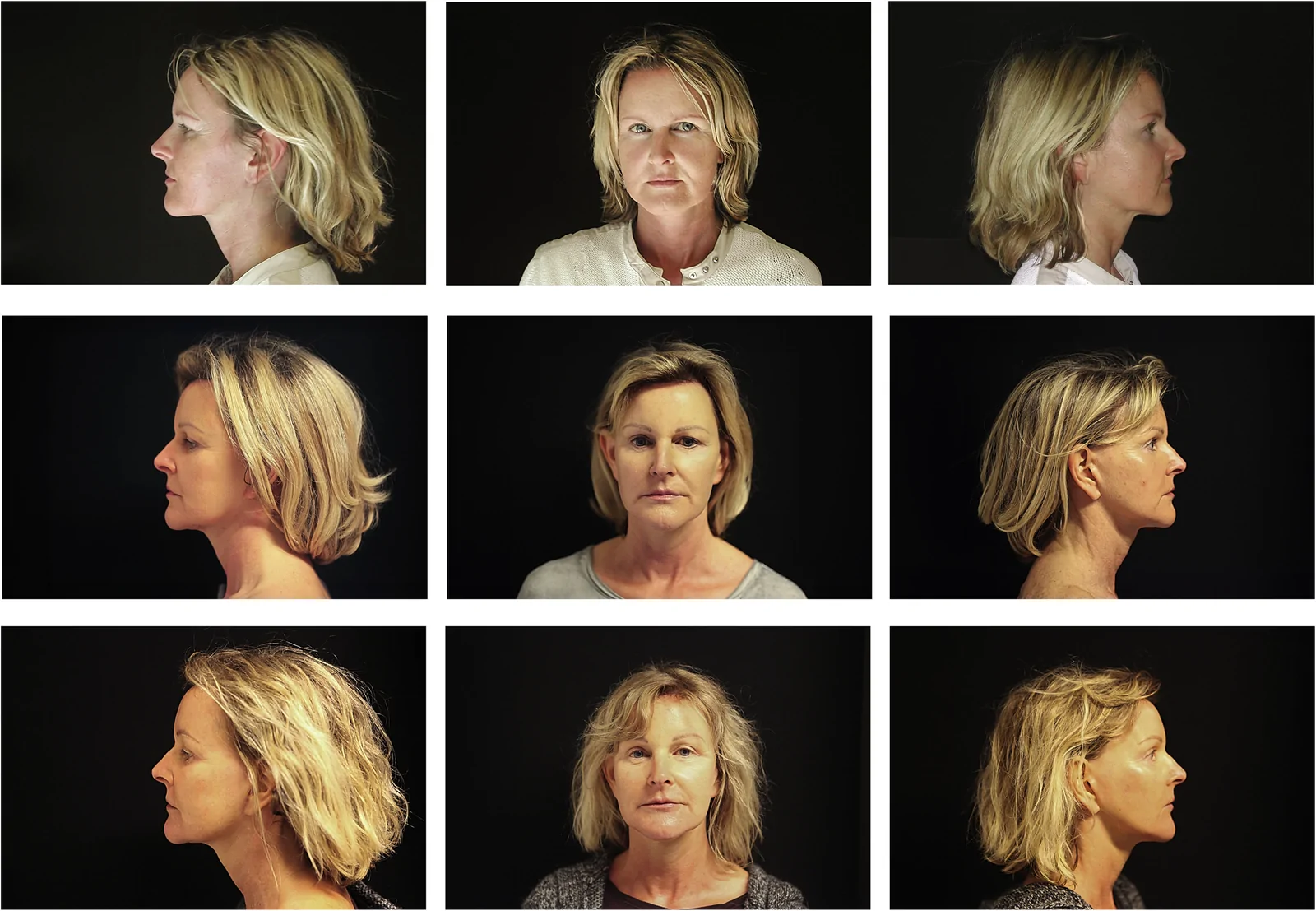
Case 2. Row 1: Age 42, 12 months postoperative primary cervicofacial lift. Row 2: Age 54, 12 months postoperative secondary cervicofacial lift and bilateral upper blepharoplasty. Row 3: Age 59, 12 months postoperative tertiary cervicofacial lift.

The reliance on clinical assessments and surgical outcomes as the primary data points may introduce some subjectivity, particularly regarding patients’ perceptions of the results. To address this, future studies should incorporate standardized Patient-Reported Outcome Measures (PROMs) to assess patients’ perceptions of their surgical outcomes in a more systematic and objective manner. This approach will provide a clearer understanding of how patients perceive their results, including factors such as satisfaction, quality of life, and psychosocial well-being.

Lastly, the longevity of the treatment was not measured except by the joint decision of the patient and the surgeon to undergo a new facelift. It should be noted that other factors, which were not accounted for in our comparison, influence this decision. These include the patients’ lifestyle, their medical history, the number of minimally invasive aesthetic procedures performed before and after each surgery, and the desires of each patient regarding their appearance. To more effectively compare the longevity of procedures, new research methodologies must be designed and applied to improve the quality of future publications.

## Case reports

Case 1 (refer to Figure 1): This patient came first at age 53 in our consultation asking for a global facial rejuvenation. A total lift was performed, it means a cervicofacial lifting combined with a brow lifting. At age 62 she sought a secondary facelift procedure, having undergone in the meantime additional medical aesthetic procedures such as injections of hyaluronic acid, lipofilling and laser treatments. Her primary concern was the laxity of the skin in the jowl area, especially on the left side (due to an asymmetric lipofilling). The surgical plan included a vertical SMAS repositioning along with a mini-liposuction of the left jawline, and a retightening of the skin of the oval. Eleven years later, at age 73, the patient returned for a tertiary procedure. Her main complaint was an overall round appearance of the face. A tertiary facial lifting was performed, in that case a skin lifting for an overall light retightening of the face, associated with a lipofilling and a blepharoplasty of the lower eyelids. All surgeries were performed by the senior author (Dr Michel E Pfulg, M.D.) but the last one (Dr Serge Lê Huu, M.D.).

Case 2 (refer to Figure 2): This patient came first in our consultation at age 41, asking for a facial rejuvenation. A primary cervicofacial lift was performed. Twelve years later, at age 53, she sought a secondary facelift procedure, having undergone in the meantime additional medical aesthetic procedures such as injections of hyaluronic acid, botulinum toxin and threads. Her primary concern was now a heavy jawline and a skin laxity in the neck. The surgical plan included a horizontal smasectomy along with a retightening of the skin to improve the oval. She also underwent a bilateral upper blepharoplasty. Five years later, at age 58, the patient returned for a tertiary facelift. All surgeries were performed by the senior author (Michel E Pfulg, M.D.).

## Conclusions

Secondary and tertiary facelift procedures are increasingly common in the treatment of aging in daily practice. Understanding the aging processes and tissue restoration following each surgical procedure is essential for achieving excellent aesthetic outcomes and optimizing patient results.

This study showed that secondary and tertiary facelifts maintain low complication rates similar to those of primary facelifts, supporting their safety and reliability. However, the durability of results from secondary procedures tends to be shorter than that of primary facelifts.

Achieving consistent and satisfactory outcomes in secondary and tertiary facelifts depends on individualized patient assessment, tailored surgical planning based on previous procedures, and meticulous intraoperative execution.

## Statement of human and animal rights, or ethical approval

This article does not contain any studies with human participants or animals performed by any of the authors. All procedures involving human participants performed in this study were in accordance with the ethical standards of the institutional and/or national research committee and the 1964 Declaration of Helsinki and its subsequent amendments or comparable ethical standards.

## Informed consent

All patients signed the general informed consent, and those whose photos were used in this publication provided specific written consent for the use of their images.

## Funding

This research did not receive any specific grant from funding agencies in the public, commercial, or not-for-profit sectors.

## Declaration of competing interest

The authors declare that they have no conflicts of interest to disclose. The authors declare that they have no affiliations with or involvement in any organization or entity with any financial interests regarding the topic or materials discussed in this manuscript.
