# Supplementary material for: Ten years of experience with secondary and tertiary cervicofacial lifting: A retrospective study of 360 procedures
Source: JPRAS Open. 2026 Mar 1;52:662–9. doi: 10.1016/j.jpra.2026.02.020 (PMC13731120; doi:10.1016/j.jpra.2026.02.020)
Supplement: Supplementary file 1 [file mmc1.docx]

**Supplementary documents:**

**Tables:**

Table 1: Cervicofacial lifting: surgical techniques utilized by the senior author:

| **Lifting/ Procedure** | **SMASectomy** | **SMAS plication** | **Neck liposuction** | **Anterior cervicoplasty** | **Total** |
| --- | --- | --- | --- | --- | --- |
| **Primary** | 211 (94%) | 14 (6%) | 81 (36%) | 3 (1%) | 225 |
| **Secondary** | 62 (70%) | 26 (30%) | 22 (25%) | 3 (3%) | 88 |
| **Tertiary** | 14 (100%) | 0 | 2 (14%) | 0 | 14 |
| **Total** | 288 (88%) | 40 (12%) | 105 (32%) | 6 (2%) | 327 |

Table 2: Quantity and type of additional procedures performed during the same surgical time as the cervicofacial lifting:

| **Associated procedures** | **Quantity (%)** | n= 327 |
| --- | --- | --- |
| Upper blepharoplasty | 103 (31%) | |
| Lipostructure | 63 (19%) | |
| Upper and lower blepharoplasty | 33 (10%) | |
| Temporal Lift | 22 (6%) | |
| Lower Blepharoplasty | 16 (5%) | |
| Rhinoplasty | 12 (4%) | |
| Genioplasty | 11 (3%) | |
| Breast augmentation | 7 (2%) | |

Table 3: Quantity, type, and incidence of complications related to cervicofacial liftings performed by the senior author:

| **Lifting/ Complications** | **Primary (n: 225)** | **Secondary (n: 88)** | **Tertiary (n: 14)** | **Total (n=327)** |
| --- | --- | --- | --- | --- |
| **Hematoma** | 2 (0.9%) | 2 (2.2%) | 0 | 4 (1.2%) |
| **Seroma** | 2 (0.9%) | 0 | 0 | 2 (0.6%) |
| **Nerve Injury** | 1 (0.4%) | 0 | 0 | 1 (0.3%) |
| **Skin Slough** | 1 (0.4%) | 0 | 0 | 1 (0.3%) |
| **Infection** | 3 (1.3%) | 1 (1.1%) | 1 (7.1%) | 5 (1.5%) |
| **Pixie ear** | 2 (0.9%) | 0 | 0 | 2 (0.6%) |
| **Total** | 11 (4.9%) | 3 (3.4%) | 1 (7.1%) | **15 (4.6%)** |

**Case Report:**

Case 3 (refer to Fig. 3): This patient came first into our consultation at age 44. She underwent a cervicofacial lift. Ten years later, at age 54, she returned asking for a new surgery. The primary concerns were sagging skin in the jowl and neck areas, along with pronounced nasolabial folds. Facial analysis also revealed that a small skin excess was remaining around the left ear lobe. The planned surgery involved a horizontal smasectomy combined with a repositioning of the skin around the ears. Nine years later, the patient returned with a recurrent ptosis of the jowls, but with a normal and very natural symmetrical aging process thanks to the previous not too aggressive surgeries; a blepharochalasis was also present on both sides. The tertiary facelift involved a conservative smasectomy and a skin redraping, paying great attention to the vectors of traction. A conservative upper blepharoplasty was also performed during this last procedure. To mention is that this patient came back for an additional lipofilling of the face, 4 years later, at age 67. All surgeries were performed by the senior author (Dr. Michel E Pfulg, M.D.).

Figure 3. Case 3:

Legends Figure 3. case 3::
 Row 1: Age 49, twelve months postoperative primary cervicofacial lift.
 Row 2: Age 59, twelve months postoperative secondary cervicofacial lift and skin redraping around the ears.
 Row 3: Age 64, twelve months postoperative tertiary cervicofacial lift and upper blepharoplasty.
